# When should factorial designs be used for late-phase randomised controlled trials?

**DOI:** 10.1177/17407745231206261

**Published:** 2023-10-31

**Authors:** Ian R White, Alexander J Szubert, Babak Choodari-Oskooei, A Sarah Walker, Mahesh KB Parmar

**Affiliations:** MRC Clinical Trials Unit at UCL, London, UK

**Keywords:** Randomised controlled trial, clinical trial, design, factorial

## Abstract

**Background:**

A 2×2 factorial design evaluates two interventions (A versus control and B versus control) by randomising to control, A-only, B-only or both A and B together. Extended factorial designs are also possible (e.g. 3×3 or 2×2×2). Factorial designs often require fewer resources and participants than alternative randomised controlled trials, but they are not widely used. We identified several issues that investigators considering this design need to address, before they use it in a late-phase setting.

**Methods:**

We surveyed journal articles published in 2000–2022 relating to designing factorial randomised controlled trials. We identified issues to consider based on these and our personal experiences.

**Results:**

We identified clinical, practical, statistical and external issues that make factorial randomised controlled trials more desirable. Clinical issues are (1) interventions can be easily co-administered; (2) risk of safety issues from co-administration above individual risks of the separate interventions is low; (3) safety or efficacy data are wanted on the combination intervention; (4) potential for interaction (e.g. effect of A differing when B administered) is low; (5) it is important to compare interventions with other interventions balanced, rather than allowing randomised interventions to affect the choice of other interventions; (6) eligibility criteria for different interventions are similar. Practical issues are (7) recruitment is not harmed by testing many interventions; (8) each intervention and associated toxicities is unlikely to reduce either adherence to the other intervention or overall follow-up; (9) blinding is easy to implement or not required. Statistical issues are (10) a suitable scale of analysis can be identified; (11) adjustment for multiplicity is not required; (12) early stopping for efficacy or lack of benefit can be done effectively. External issues are (13) adequate funding is available and (14) the trial is not intended for licensing purposes. An overarching issue (15) is that factorial design should give a lower sample size requirement than alternative designs. Across designs with varying non-adherence, retention, intervention effects and interaction effects, 2×2 factorial designs require lower sample size than a three-arm alternative when one intervention effect is reduced by no more than 24%–48% in the presence of the other intervention compared with in the absence of the other intervention.

**Conclusions:**

Factorial designs are not widely used and should be considered more often using our issues to consider. Low potential for at most small to modest interaction is key, for example, where the interventions have different mechanisms of action or target different aspects of the disease being studied.

## Background

In a factorial design for a randomised controlled trial (RCT), two or more randomised comparisons are carried out independently in the same sample of patients.^1^ This design has the potential to address multiple questions in an efficient way. For example, a 2×2 factorial design compares each of two interventions (A, B) to control by randomising participants to control, A-only, B-only or both A and B; we call each of these four randomised combinations an arm. The analysis then compares all those randomised to A (with or without B) to all those not randomised to A (with or without B), and similarly for B. Compared to two 2-arm RCTs (A versus control and B versus control) or a three-arm RCT (A versus B. versus control), a 2×2 factorial RCT comparing A to control, and B to control, often requires fewer participants and hence resources.

Factorial designs are generally used in late-phase trials. For example, the trial of imaging and surveillance in seminoma testis (TRISST) trial considered whether men with successfully treated testicular cancer could safely have a lighter surveillance schedule.^2^ Men were randomised to 3 scans or the standard 7 scans over 5 years, and also to magnetic resonance imaging (MRI) scans or the standard computed tomography (CT) scans. Separate analyses addressed the non-inferiority of 3 scans versus 7 and MRI versus CT scans.

Extended factorial designs are possible and allow more questions to be addressed simultaneously. The transfusion and treatment of severe anemia in African children trial (TRACT) trial was a 3×2×2 factorial in which children admitted to the hospital with severe anaemia were randomised to (1) liberal blood transfusion, conservative transfusion or no transfusion, (2) post-discharge multi-vitamin supplementation or routine care and (3) post-discharge cotrimoxazole prophylaxis or no prophylaxis.^3^

We use the term ‘factorial randomisation’ to mean a randomisation to all possible intervention combinations, ‘factorial analysis’ to mean an analysis comparing all those randomised to an intervention (alone or with other interventions) with all those randomised to the corresponding control (alone or with other interventions), and ‘factorial design’ to mean a trial with both factorial randomisation and factorial analysis. A ‘partial factorial design’ is a factorial design where some participants do not participate in some randomisations or are randomised between a reduced set of interventions: for example, children with severity signs in the TRACT trial were not eligible for randomisation to no transfusion.^3^ We consider factorial designs aiming to evaluate each intervention separately; larger sample sizes are needed to assess, for example, whether the combined intervention is more effective than either individual intervention.^4,5^

Factorial designs are sensitive to interactions between interventions, an issue we discuss below. Because of this, factorial randomisation is sometimes used without factorial analysis. This is appropriate where the aim is to explore interactions between interventions^1,6^ and/or an interaction between interventions is a real possibility. It is also appropriate where the aim is to compare each intervention combination to control. An example is the systemic therapy for advanced or metastatic prostate cancer: evaluation of drug efficacy (STAMPEDE) trial in oncology, where the initial aim was to compare each of zoledronic acid, celecoxib, docetaxel, zoledronic acid plus docetaxel and celecoxib plus docetaxel with control.^7^ Factorial analysis was avoided because ‘there is no good evidence to support the notion that the intervention effects would only be additive at the patient level and we cannot rule out other forms of interaction: synergy, antagonism and a ceiling effect’.^8^ Finally, factorial randomisation without factorial analysis can be used where the aim is to determine which intervention combination is the best.^9–11^

Factorial trials have started to be used more, particularly in the field of infections where the different interventions are typically in different ‘domains’ of intervention.^12,13^ However, we believe that factorial designs may be an under-used tool for addressing multiple questions at little extra difficulty or cost. For example, we searched a registry of RCTs, clinicaltrials.gov, to identify all phase III/IV interventional studies with randomised allocation first posted to the registry from 1 January 2015 to 31 July 2022 (search done 2 August 2022). Each clinicaltrials.gov record including the word ‘factorial’ was reviewed by AS/IW to determine whether it truly represented a factorial design. Reasons why trials might be reported as factorial but determined as non-factorial included an A/B/A + B design with no control group, and a 2-way randomisation across two subgroups where the subgroup variable was not randomised. Of 22,403 phase III/IV interventional studies with randomised allocation, 206 (0.9%) were factorial. Factorial trials constituted a smaller fraction (0.14%, 9/6212) of trials wholly funded by industry than of other trials (1.22%, 197/16,191, *p* < 0.001 for difference).

We therefore aimed to compile a checklist of issues to consider when choosing between a factorial design and other designs, based on a narrative review of literature relating to the design of factorial RCTs.

## Methods

### Narrative review of design of factorial RCTs

The full text of articles published in the following methodological journals between 1 January 2000 and 31 July 2022 was searched (5 April 2019, 11 August 2022) for ‘factorial’: Biometrics, BMC Medical Research Methodology, Clinical Trials, Journal of the Royal Statistical Society (all series), Pharmaceutical Statistics, Statistics in Medicine, Trials and Statistical Methods in Medical Research. PubMed was also searched (26 April 2019, 11 Aug 2022) with the same date ranges to identify citations with ‘factorial’ in the title (articles relating specifically to designing factorial RCTs were considered likely to include this). Titles, relevant abstracts and then the full text of relevant articles were reviewed by AS/IW to identify articles relating to designing factorial RCTs, including less recent key references in relevant articles. Articles relating to phase II and cluster-randomised RCTs were excluded, as, in general, were articles relating to the protocol or results of a specific RCT. Issues to consider were identified based on these and the authors’ personal experience (including previous work^2,3,14–16^ and ongoing trials).

### Sample size required for factorial versus other designs

A key potential advantage of a factorial design is its ability to answer multiple questions with very little, if any, increase in total size. However, this depends on a number of issues including the assumption of no substantial interaction. In order to assess the gains in efficiency in practice, we compared the sample size required to show superiority of one intervention (A) over control using factorial and other designs under various scenarios.

Specifically, we computed the required sample sizes to give 90% power for showing superiority of A compared with control in a 2×2 factorial design, assuming a 5% two-sided significance level. The four arms are described as 0, A, B and AB. We assumed values for each arm for: the expected outcome with perfect adherence (taking A to be effective, allowing B to be effective or ineffective, and varying the interaction between A and B); the expected proportion of non-adherers, assumed for sample size purposes to have the same outcome mean as the control arm; and the expected proportion of missing outcome data, assumed to be excluded from the analysis. We allowed unequal allocation ratios. We calculated the relative efficiency of evaluating A versus control in a factorial design compared with a corresponding three-arm trial (A versus B versus control) as the inverse ratio of sample sizes required to achieve the same power. Sample size formulae are given in the supplemental material.

Initially, we assumed that the outcome is quantitative and that B is ineffective. The actual sample size and outcome variance did not affect the relative efficiency results. We considered the following scenarios:

1. Base case: all arms have 10% missing data and 10% non-adherence among observed data; equal allocation.
2. Perfect case: no missing data, perfect adherence, equal allocation.
3. Double missing with A: like base case, but arms A and AB have 20% missing data.
4. Double missing with B: like base case, but arms B and AB have 20% missing data.
5. Double non-adherence with A: like base case, but arms A and AB have 20% non-adherence.
6. Double non-adherence with B: like base case, but arms B and AB have 20% non-adherence.
7. Double controls: like base case, but twice as many participants are allocated to the control arm as to each other arm. (i.e. 4:2:2:1 for factorial and 4:2:2 for three-arm).

We then repeated this set of calculations three times. In set 2, the outcome was again quantitative, but intervention B was effective. This gave the same results as for set 1, except that scenario 6 lost efficiency (results not shown). In sets 3 and 4, the outcome was binary, with a proportion of 40% in the control arm expected to reduce to 24% in A arm with perfect adherence (risk ratio = 0.6). In set 3, B was ineffective, and in set 4, B was as effective as A.

We measured the interaction as a percentage of the main effect of A and varied it from 0% to 100%. For binary outcome, this was done on the risk difference scale (other scales are possible).

## Results

Twenty-seven articles relating to designing factorial RCTs were identified, including 14 in methodological journals^1,5,9,17–30^ and 13 in clinical journals^4,31–43^ of which some were overview articles. Based on these articles and the authors’ personal experience, issues to consider when planning a factorial RCT were identified.

### Clinical issues

#### Co-administration

Most factorial RCTs require co-administration of interventions. Factorial RCTs are more desirable if co-administration is easy. Difficulty of co-administration could be a reason to reject a factorial design out of hand.

#### Safety

Factorial RCTs are more desirable if the risk of safety issues from co-administration, above the individual risks of the separate interventions, is low.

#### Combination intervention

Factorial RCTs are more desirable if initial or additional safety or clinical data are wanted on the combination intervention. Safety data could be valuable to explore the risk of drug interactions, but sample sizes in a factorial design might not be adequate for a full safety evaluation of the combination intervention. Clinical data could include pharmacokinetic or pharmacodynamic data.

#### Interaction (effect modification)

Factorial RCTs are more desirable if the potential for interaction is low: that is, if the effect of one intervention is unlikely to differ substantially when another intervention is also administered. It is sometimes stated that the factorial analysis rests on a no-interaction assumption: for example, ICH E9 Statistical Principles for Clinical Trials recommends that evidence that there is likely to be no interaction is established in advance using prior information and data.^6^ However, it is more useful to accept that interactions may occur and consider their plausibility, potential size and impact.

Interactions are less likely if the different interventions target different underlying domains of intervention:^33^ for example, in the TRACT trial above, blood transfusion, anti-bacterial prophylaxis and nutritional support are unlikely to interact.^3^ Similarly the reduction of early mortality in HIV infected adults and children starting antiretroviral therapy (REALITY) trial assessed the impact of interventions to reduce HIV viral load faster, reduce risks of co-infections and improve nutritional status on mortality in those with advanced HIV starting treatment.^16^

Factorial designs usually have little power to detect interaction, but the suspicion of interaction has negative consequences for the interpretation of the trial. This is because the factorial analysis estimates the average intervention effect in a population randomly assigned to the other interventions, but in the presence of interaction, this is rarely the estimand of clinical interest. Instead, clinical interest usually lies in the average intervention effect in a population either *receiving* or *not receiving* the other interventions; for example, if the other intervention B is found to be effective, then clinical interest is more likely to be in a population *receiving* intervention B.^28^

Interaction can also reduce the power of a factorial design if interventions are less effective in the presence of other interventions:^44^ we explore this below, and revisit the interaction issue in the discussion.

Health economists have suggested that interaction may be more likely to occur for costs and quality-adjusted life years, and may therefore have a larger impact on health economic evaluations.^25,26^

#### Balancing other interventions

In situations where there is no ‘standard of care’ for the alternative intervention(s), these may be used or not used depending on physician preference, and hence, in an open-label trial, may be unbalanced across other comparisons. A factorial randomisation forces the alternative intervention(s) to be balanced across groups of the intervention of interest and may be more desirable if the estimand of interest is a pure intervention comparison.

#### Eligibility criteria

Factorial RCTs are more desirable if eligibility criteria for the different comparisons are similar. If eligibility criteria are different, then requiring all participants to be eligible for all comparisons would reduce the pool of potential participants; an alternative is a partial factorial design, where participants are included if they are eligible for any one or more comparisons, and are randomised in all eligible comparisons.^30^

### Practical issues

#### Recruitment

Factorial RCTs are less desirable if they compromise recruitment. For example, if there is a clinician or patient preference against one particular intervention, then including that intervention in a factorial design may adversely affect recruitment. On the other hand, addressing a number of different clinical questions could improve recruitment by increasing the enthusiasm of both patients and investigators for a factorial trial.

#### Adherence

Factorial RCTs are less desirable when one or more of the interventions (or any toxicities associated with it) is likely to reduce adherence to other interventions, since this will reduce the effectiveness of the other intervention and reduce power.

#### Retention

Factorial RCTs are less desirable when one or more of the interventions is likely to reduce retention in the study, since this will increase the amount of missing data for all comparisons and hence reduce power and increase concerns about bias.

Where one intervention may adversely affect recruitment, adherence or retention, it may be preferable to explore that intervention in a separate trial where these complex issues can be specifically addressed without impacting on other comparisons.

#### Blinding

Factorial RCTs are more desirable if blinding is easy to implement (or not required). In a 2-arm RCT, it is often feasible to manufacture a placebo with similar physical properties (including size, colour, taste and smell) to the active medicine. In a 2×2 factorial RCT, one option is to use A + B, A + placebo B, B + placebo A and placebo A + placebo B (‘double-dummy’). However, this might be undesirable because the increased number of pills makes non-adherence more likely. To minimise pill burden, it may be preferable to use combination pills containing A + B, A-only, B-only or placebo. However, this might not be feasible, for example, if interventions are given at different frequencies (e.g. A once daily in the morning versus B twice daily). It might also be undesirable because participants cannot stop just one of the interventions.

### Statistical issues

#### Scale of analysis

Factorial RCTs are more desirable if a suitable scale can be identified on which interaction is unlikely. For example, for binary outcomes, lack of interaction on the odds ratio scale is not the same as the lack of interaction on the risk ratio scale.^45^ Interactions tend to be less common on the odds ratio and risk ratio scales than on the risk difference scale.^46^ Once a suitable scale has been identified, the analysis (and in particular assessment of interactions) must be performed on that scale.

#### Multiplicity

Factorial RCTs are less desirable if adjustment for multiplicity is required. A standard 2-arm RCT would typically be designed with a two-sided false-positive (type I) error rate of 5%, and two independent 2-arm RCTs would have an overall false-positive error rate of nearly 10%. A factorial RCT may be viewed as two 2-arm RCTs, and therefore, an error rate of 5% per comparison may be considered acceptable, or alternatively, control of the overall error rate may be considered necessary. If different scientific questions are being addressed, particularly with different primary endpoints, then adjustment for multiplicity should not be required^47^ but has been the topic of debate.^4,21,22,31^

#### Stopping

Factorial RCTs are less desirable if adequate ways cannot be found to stop comparisons early, either for efficacy or for lack of benefit, without damaging other ongoing comparisons. We recommend using standard statistical stopping guidelines that control the pairwise type 1 error rate for each comparison using factorial analysis,^30^ recognising that statistical guidelines are only part of the decision to stop a trial arm early.

### External issues

#### Funders

Factorial RCTs are more desirable if adequate funding is available. Even if they are an efficient way to answer multiple questions, factorial designs may still be more expensive than a single two-arm trial, and funders may conservatively prefer the latter, depending on the scientific importance of the additional questions. However, funders increasingly prefer platform trials for their increased efficiency,^48^ and factorial trials share with platform trials an ability to test many interventions within a single protocol.

#### Regulators

Factorial RCTs are not typically used for licensing trials, where the focus is usually instead on pre-specification of other interventions, making the estimand more clear-cut.

### Other issues

The literature review raised the concern that complex factorial designs with small sample sizes can lead to larger covariate imbalances across arms than simpler designs, and therefore, minimisation procedures may be preferable to other randomisation schemes.^29^ This point requires attention in some small trials but does not affect the desirability of a factorial design.

### Overarching issue: sample size

The issues described above are summarised in Table 1, together with the overarching issue of sample size. The main rationale for factorial RCTs is that they can have a lower sample size than alternative designs, including two independent 2-arm RCTs or 3-arm RCTs. However, sample size requirements may be increased by a number of the issues considered above, in particular interaction, adherence and retention.^19,44^

**Table 1.** Key issues to consider around factorial designs.

Issue to consider	A factorial design is more desirable if
Clinical issues	
Co-administration	Interventions can be easily co-administered.
Safety	Risk of safety issues from co-administration, above individual risks of the separate interventions, is low.
Combination intervention	Safety or clinical data are wanted on the combination intervention.
Interaction (effect modification)	Effect of each intervention is unlikely to be substantially different in the presence of the other intervention(s).
Balancing other interventions	Other interventions are likely to be unbalanced if not randomised
Eligibility criteria	Eligibility criteria for all comparisons are similar.
Practical issues	
Recruitment	Recruitment is not harmed by including many interventions.
Adherence	Each intervention and the toxicities associated with it is unlikely to reduce adherence to (and hence effectiveness of) the other intervention.
Retention	Each intervention is unlikely to reduce overall follow-up.
Blinding	Blinding is easy to implement or not required.
Statistical issues	
Scale of analysis	A suitable scale of analysis can be identified on which interaction is unlikely.
Multiplicity	Adjustment for multiplicity is not required.
Stopping	Adequate ways can be found to stop comparisons early for efficacy or lack of benefit.
External issues	
Funders	Funding is adequate for the complexity of the design.
Regulators	The trial is not intended for licensing purposes.
Overarching issue	
Sample size	Factorial design gives a lower sample size requirement than alternative designs.

Sample size in factorial RCTs may also need inflation to allow for multiple interventions being effective.^49^ With a survival-type endpoint, the power depends on the number of events (e.g. deaths). For a factorial RCT with the same time-to-event outcome for each comparison (or correlated time-to-event outcomes, such as, overall and progression-free survival), one intervention being effective decreases the number of events available for the other comparisons. The sample size might need to be inflated to allow for this. For a binary endpoint, whether sample size inflation is necessary depends on the proportions hypothesised under the null and alternative hypotheses and on the anticipated effect of the combined intervention.

**Figure 1.**
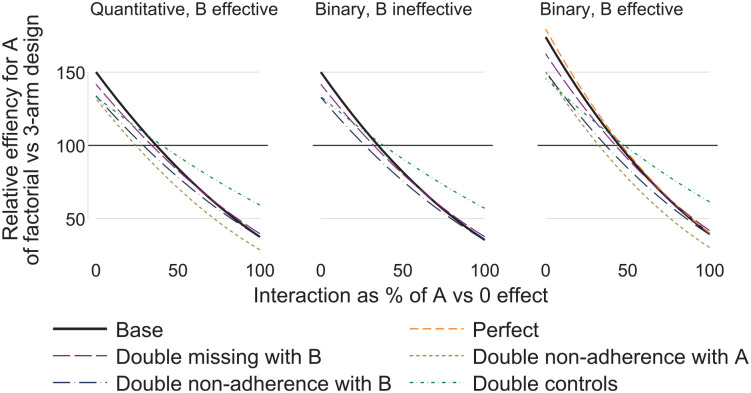
Relative efficiency of factorial versus three-arm design graphed against interaction, by outcome type (quantitative or binary) and whether B is effective.

We illustrate how to take account of interaction, adherence, retention and efficacy of other interventions when comparing the sample size required for a factorial design with alternative designs. Figure 1 shows the relative efficiency of the factorial design compared with the three-arm design for estimating the effect of intervention A. The panels represent outcome type (quantitative or binary) and whether B is effective. The panel for quantitative outcome and B ineffective is not shown, as the results are the same as for quantitative outcome and B effective, except that ‘double non-adherence with A’ is the same as the base case. Results for ‘Double missing with A’ are not shown, as they were the same as for the base case. Results for ‘Perfect’ and ‘Double missing with B’ are not shown in some panels where their results were the same as for the base case. The supplemental material gives the numerical results.

Comparing scenarios within panels of Figure 1, we see that missing data with A affects factorial and three-arm designs equally, while missing data with B and non-adherence with B only affect the factorial design. Non-adherence with A affects both designs equally if B is ineffective, but affects the factorial design more if B is effective (because AB non-adherers lose both intervention effects). Doubling controls moves all relative efficiencies towards 100%.

Comparing panels in Figure 1, the main finding is that the effectiveness of B on a binary outcome improves the efficiency of the factorial design for low interactions. This is because low interactions make the risks smaller and hence the variances smaller; this change in variance does not occur with a quantitative outcome.

In the base case for a quantitative outcome, the factorial design is superior when the interaction is less than 37% of the effect of A in the absence of B (i.e. A versus 0 effect). In the other scenarios, this figure of 37% ranges from 24% to 48% (Table 2).

**Table 2.** Critical value of interaction (expressed as % of A versus 0 effect on difference scale) above which factorial design becomes less efficient than three-arm design.

Outcome	Quantitative		Binary	
Nature of intervention B	Ineffective	Effective	Ineffective	Effective
Base case	37	37	35	45
Perfect case	37	37	35	46
Double missing with A	37	37	35	45
Double missing with B	34	34	32	43
Double non-adherence with A	37	24	35	32
Double non-adherence with B	29	29	27	37
Double controls	40	40	38	48

## Conclusion

Many factorial RCTs have been conducted, but they still account for less than 1% of all RCTs, suggesting that there may be scope for their greater use given increased emphasis on efficiency in trial design. We have therefore proposed a number of issues to consider when deciding between factorial and other designs.

Factorial RCTs should be considered as alternatives to multi-arm RCTs, and as extensions to standard 2-arm RCTs, particularly when there may be an opportunity to address additional management questions. A particular strength of factorial designs is assessing multiple different domains of intervention for one condition, where interaction is *a priori* much less likely, and additional scientific questions can be addressed in many cases for free, or nearly for free. The TRACT and REALITY trials are good examples of this kind of approach, assessing multiple different underlying mechanisms to improve outcomes from severe anaemia and HIV.^3,16^ Other examples are the large platform trials randomized embedded multifactorial adaptive platform for community-acquired pneumonia (REMAP-CAP) and *Staphylococcus aureus network adaptive platform trial* (SNAP), which have partial factorial interventions in multiple different domains of treatment for pneumonia in the intensive care unit (e.g. antibiotic choice, duration, adjunctive macrolides, corticosteroids) and *Staphylococcus aureus* bacteraemia.^12,13^

Of the issues discussed, interaction is often seen as the largest problem with factorial designs, with consequences for the trial’s power and interpretability. At the trial design stage, investigators should assess the possible magnitude and likelihood of an interaction. Often, this relies on clinical judgement since prior data can seldom reliably inform interaction size. We suggest that the impact of plausible interactions is best explored by their consequences for the trial’s power since this can be precisely quantified using the methods described here. If important interaction is plausible then alternative designs should be considered including (in the 2×2 setting) a 2-arm design (ignore one intervention), a 3-arm design (omit the combined intervention), a 4-arm design (using a non-factorial analysis)^9^ or variants on the factorial analysis.^5,50^

A finding of unexpected interaction complicates the interpretation and may damage the credibility of a factorial trial. In some cases this will represent genuine complexity which would not have been discovered with a simpler design; in other cases, it may be a chance finding. If a *quantitative interaction* is observed, where the estimated effects of A with and without B both suggest benefit from A, but of different magnitude, then in practice, the impact on inference may be small. If a *qualitative interaction* is observed,^51^ where there is some statistical evidence that A has benefit if given without B, but not with B, or even more importantly that it is harmful with B, then this is important knowledge which would never have been uncovered without a factorial design. One of the most relevant factors to consider may be whether the other intervention, B, is already being used in clinical practice. The best approach is to set out a clear statistical analysis plan which specifies (1) when a factorial analysis will be abandoned: if interactions are a priori unlikely then it may be appropriate to pre-specify a strong statistical significance level (e.g. *p* < 0.01) here; and (2) what alternative analysis will be used, taking account of the clinical setting, if a factorial analysis is abandoned.

We showed that a factorial design has greater power than a 3-arm design when the interaction is less than about 30–40% of the main effect. This needs to be assessed at the trial design stage. Assessing it from trial results is usually unhelpful (because sampling variation is large) and should not be used to argue retrospectively that a factorial design was inappropriate. Future research could explore empirical evidence about interaction sizes in finished trials, using meta-analysis methods to remove sampling variation.

Limitations of our study include that our issues to consider are derived from our literature review and our experience and not from a Delphi survey. Our sample size comparisons used a broad but by no means exhaustive set of scenarios; slightly better results for the three-arm design could have been achieved by using unequal allocation ratios.

In summary, the issues described should be considered when designing factorial RCTs. This applies both for the primary and secondary endpoints including patient-reported outcomes and cost-effectiveness.^25,26^

## Supplemental Material

sj-docx-1-ctj-10.1177_17407745231206261 – Supplemental material for When should factorial designs be used for late-phase randomised controlled trials? Supplemental material, sj-docx-1-ctj-10.1177_17407745231206261 for When should factorial designs be used for late-phase randomised controlled trials? by Ian R White, Alexander J Szubert, Babak Choodari-Oskooei, A Sarah Walker and Mahesh KB Parmar in Clinical Trials
