# Supplementary material for: When should factorial designs be used for late-phase randomised controlled trials?
Source: Clin Trials. Author manuscript; Available in PMC 2024 Apr 9. (PMC7615816; doi:10.1177/17407745231206261)
Supplement: Supplementary Material [file EMS194375-supplement-Supplementary_Material.docx]

SUPPLEMENTAL MATERIAL

When should factorial designs be used for randomised controlled trials?

## Sample size approach

For a participant randomised to A ($a=1$) or not ($a=0$) and to B ($b=1$) or not ($b=0$), we define:

$\mu_{ab}=$ the mean outcome (for a quantitative outcome) or the outcome probability (for a binary outcome) if participants perfectly adhered;

$\gamma_{ab}=$ the adherence probability;

$\rho_{ab}=$ the response probability (i.e. the probability of the outcome not being missing);

$\nu_{ab}=$ a number proportional to the number randomised.

We allow for interaction by not requiring any relationship between $\mu_{00},\mu_{01},\mu_{10}$ and $\mu_{11}$.

Allowing for non-adherence, which is assumed to lead to mean outcome $\mu_{00}$, the observed mean outcome is

$$\mu_{ab}^{*}=\gamma_{ab} \mu_{ab}+\left( 1-\gamma_{ab} \right)\mu_{00}.$$

We will compute the sample size for both designs in terms of the properties of the two groups compared for the A vs. no-A comparison: their means $\tilde{\mu}_{1},\tilde{\mu}_{0}$ and the numbers $\tilde{\nu}_{0},\tilde{\nu}_{1}$ of individuals with observed outcomes as fractions of the total number randomised.

### Factorial design

Allowing for non-response, the sample size of the analysed data in the $a, b$ arm as a fraction of the total number randomised is

$$\frac{\nu_{ab}\rho_{ab}}{\nu_{++}}.$$

Hence for all $A=a$ (i.e. combining those allocated to B and not allocated to B), the observed mean outcome is

$$\tilde{\mu}_{a}=\frac{\sum_{b} \nu_{ab} \rho_{ab}\mu_{ab}^{*}}{\sum_{b} \nu_{ab} \rho_{ab}}=\frac{\nu_{a0} \rho_{a0} \mu_{a0}^{*}+\nu_{a1} \rho_{a1} \mu_{a1}^{*}}{\nu_{a0} \rho_{a0} +\nu_{a1} \rho_{a1}}$$

and the analysed data as a fraction of all those randomised is

$$\tilde{\nu}_{a}=\frac{\sum_{b} \nu_{ab}\rho_{ab}}{\nu_{++}}=\frac{\nu_{a0}\rho_{a0}+\nu_{a1}\rho_{a1}}{\nu_{00}+\nu_{01}+\nu_{10}+\nu_{11}}.$$

### Three-arm design

The alternative design omits the AB arm in the design and omits the B arm in estimating the effect of A, so $\tilde{\mu}_{a}$ now refers to the mean for the A arm ($a=1$) and the 0 arm ($a=0$), and $\tilde{\nu}_{a}$ now refers to the analysed data in those arms as a fraction of all those randomised (i.e. all those randomised to 0, A or B). We use the same notation so that participants are randomised to 0, A, B in the ratio $\nu_{00}:\nu_{10}:\nu_{01}$. The observed mean outcomes are

$\tilde{\mu}_{1}=\mu_{10}^{*}$ and $\tilde{\mu}_{0}=\mu_{00}^{*}$

and the sample sizes of the analysed data as a fraction of all those randomised are

$\tilde{\nu}_{1}=\frac{\nu_{10}\rho_{10}}{\nu_{00}+\nu_{10}+\nu_{01}}$ and $\tilde{\nu}_{0}=\frac{\nu_{00}\rho_{00}}{\nu_{00}+\nu_{10}+\nu_{01}}$.

### Both designs

The anticipated intervention effect for A is

$${\delta=\tilde{\mu}}_{1}-\tilde{\mu}_{0}.$$

Implicit in this expression is an assumption that analysis will not adjust for randomisation to B, which is valid if randomisation to A and randomisation to B are independent in the observed data (which is true in all our examples).

We next express the variance of the estimated intervention effect as $\frac{V}{N}$ where $N$ is the total number randomised. For quantitative data,

$$V=\sigma^{2}\left( \frac{1}{\tilde{\nu}_{1}}+\frac{1}{\tilde{\nu}_{0}} \right).$$

We approximate the within-group variance $\sigma^{2}$ to be the same in both designs, although the groups in the factorial design include a mixture of participants randomised to B or not, while the groups in the three-arm design include no B. This approximation is valid when the effect of B is small compared to the standard deviation $\sigma$.

For binary data, the variance is different under the null ($V_{null}$) and under the alternative ($V_{alt}$):

$$V_{alt}=\frac{\tilde{\mu}_{1}\left( 1-\tilde{\mu}_{1} \right)}{\tilde{\nu}_{1}}+\frac{\tilde{\mu}_{0}\left( 1-\tilde{\mu}_{0} \right)}{\tilde{\nu}_{0}}$$

$$V_{null}=\tilde{\mu}_{.}\left( 1-\tilde{\mu}_{.} \right)\left\{ \frac{1}{\tilde{\nu}_{1}}+\frac{1}{\tilde{\nu}_{0}} \right\}$$

where $\tilde{\mu}_{.}$ is the overall mean in the analysed data,

$$\tilde{\mu}_{.}=\frac{\sum_{a} \tilde{\nu}_{a} \tilde{\mu}_{a}}{\sum_{a} \tilde{\nu}_{a}}.$$

To have power $\beta$ to reject the null at one-sided significance level $\alpha$, we require a sample size given by the formulae

$$N=\frac{V\left( z_{\alpha}+z_{\beta} \right)^{2}}{\delta^{2}}$$

for continuous data and

$$N=\frac{\left( \sqrt{V_{null}} z_{\alpha}+\sqrt{V_{alt}} z_{\beta} \right)^{2}}{\delta^{2}}$$

for binary data.

Finally, letting the sample sizes computed for the factorial and three-arm designs be $N_{fac}$ and $N_{3arm}$ respectively, the relative efficiency of the factorial design is

$$RE=\frac{N_{3arm}}{N_{fac}}.$$

## Numerical results

Table. Relative efficiency (%) of factorial vs. three-arm design. Interaction is expressed as % of A vs. 0 effect on difference scale. [Red: the same or almost the same as other scenarios.]

| Nature of intervention B | Ineffective | | | | | | | | | Effective | | | | | | |
| --- | --- | --- | --- | --- | --- | --- | --- | --- | --- | --- | --- | --- | --- | --- | --- | --- |
| Interaction % | 0 | 25 | | 50 | | 75 | | 100 | | 0 | | 25 | | 50 | 75 | 100 |
| *Quantitative outcome* |  |  | |  | |  | |  | |  | |  | |  |  |  |
| Base case | 150 | | 115 | | 84 | | 59 | | 38 | 150 | 115 | | 84 | | 59 | 38 |
| Perfect case | 150 | | 115 | | 84 | | 59 | | 38 | 150 | 115 | | 84 | | 59 | 38 |
| Double missing with A | 150 | | 115 | | 84 | | 59 | | 38 | 150 | 115 | | 84 | | 59 | 38 |
| Double missing with B | 142 | | 110 | | 83 | | 59 | | 40 | 142 | 110 | | 83 | | 59 | 40 |
| Double non-adherence with A | 150 | | 115 | | 84 | | 59 | | 38 | 132 | 99 | | 71 | | 47 | 29 |
| Double non-adherence with B | 134 | | 104 | | 78 | | 56 | | 38 | 134 | 104 | | 78 | | 56 | 38 |
| Double controls | 133 | | 112 | | 93 | | 75 | | 59 | 133 | 112 | | 93 | | 75 | 59 |
| *Binary outcome* |  | |  | |  | |  | |  |  |  | |  | |  |  |
| Base case | 150 | | 113 | | 82 | | 56 | | 35 | 174 | 130 | | 93 | | 63 | 40 |
| Perfect case | 150 | | 113 | | 81 | | 56 | | 35 | 179 | 133 | | 95 | | 64 | 40 |
| Double missing with A | 150 | | 113 | | 82 | | 56 | | 35 | 174 | 130 | | 93 | | 63 | 40 |
| Double missing with B | 142 | | 109 | | 80 | | 57 | | 38 | 162 | 124 | | 91 | | 64 | 42 |
| Double non-adherence with A | 150 | | 113 | | 82 | | 56 | | 36 | 150 | 110 | | 78 | | 51 | 30 |
| Double non-adherence with B | 133 | | 102 | | 76 | | 54 | | 35 | 150 | 115 | | 84 | | 59 | 39 |
| Double controls | 133 | | 111 | | 91 | | 73 | | 57 | 146 | 121 | | 98 | | 79 | 61 |
